# Ubiquitination Enzymes in Cancer, Cancer Immune Evasion, and Potential Therapeutic Opportunities

**DOI:** 10.3390/cells14020069

**Published:** 2025-01-07

**Authors:** Aiman B. Awan, Maryiam Jama Ali Osman, Omar M. Khan

**Affiliations:** 1 College of Health and Life Sciences, Hamad Bin Khalifa University, Doha P.O. Box 34110, Qatar; aibu38539@hbku.edu.qa (A.B.A.); maos34113@hbku.edu.qa (M.J.A.O.); 2 Research Branch, Sidra Medicine, Doha P.O. Box 34110, Qatar

**Keywords:** ubiquitination, cell cycle, E3 ligases, oncogenes, tumor suppressor, PROTACs, cancer, immune evasion

## Abstract

Ubiquitination is cells’ second most abundant posttranslational protein modification after phosphorylation. The ubiquitin–proteasome system (UPS) is critical in maintaining essential life processes such as cell cycle control, DNA damage repair, and apoptosis. Mutations in ubiquitination pathway genes are strongly linked to the development and spread of multiple cancers since several of the UPS family members possess oncogenic or tumor suppressor activities. This comprehensive review delves into understanding the ubiquitin code, shedding light on its role in cancer cell biology and immune evasion. Furthermore, we highlighted recent advances in the field for targeting the UPS pathway members for effective therapeutic intervention against human cancers. We also discussed the recent update on small-molecule inhibitors and PROTACs and their progress in preclinical and clinical trials.

## 1. Introduction

Ubiquitination is a reversible covalent protein modification first discovered ~26 years ago [1,2]. It is an addition of an ~8.5 kDa ubiquitin protein on another protein that typically occurs on the acceptor lysine of the substrate protein. Although non-lysine and non-proteinaceous ubiquitination is also possible and is discussed in detail elsewhere [3]. Largely, ubiquitination serves as a signal for proteasome-mediated protein degradation [4]. However, non-degradative ubiquitination also occurs and is responsible for controlling diverse cellular functions [4].

**Figure 1:**
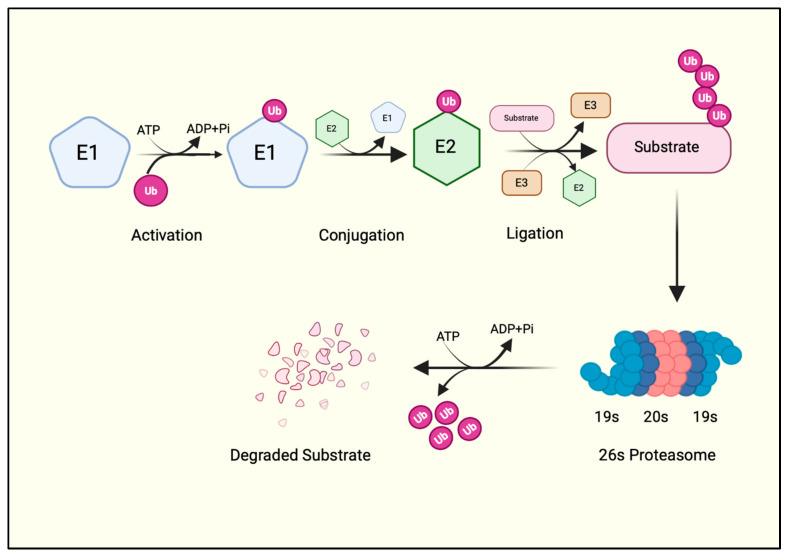
The ubiquitination cascade. A ubiquitin molecule is first charged by the E1 enzyme at the expense of an ATP molecule. The charged ubiquitin is then transferred to the E2 via a transthiolation reaction. E2 collaborates with E3 ubiquitin ligase, which adds ubiquitin to the protein substrate. A polyubiquitinated substrate is recognized by the proteasome machinery, where the substrate is degraded, and ubiquitin is recycled. Created with BioRender.com.

Ubiquitination is one of the most abundant and evolutionarily conserved posttranslational protein modifications, so much so that all proteins undergo at least one ubiquitination event during their lifecycle [4]. It is a multi-step process carried out in a sequential manner by ubiquitin-activating (E1), ubiquitin-conjugating (E2), and ubiquitin ligase enzymes (E3)—collectively regarded as the ubiquitin–proteasome system (UPS) (Figure 1). The UPS is a tightly regulated system that breaks down intracellular proteins. It controls various cellular processes, including cell cycle control, DNA damage repair, signal transduction, immune response, and response to oxidative stress.

First, an E1 enzyme forms a thioester bond with ubiquitin between the C-terminal glycine (Gly76) of ubiquitin and the active cysteine (Cys) of the E1 enzyme in an ATP-dependent manner. A transesterification reaction then transfers the activated ubiquitin to the Cys residue of the E2 enzyme and forms an E2-ubiquitin thioester intermediate. The E2-ubiquitin thioester moiety then requires an E3 ligase enzyme to finally add the ubiquitin to the protein substrate [4] (Figure 1). Thus, E3 ligases are the substrate-specific component of the UPS that catalyzes the covalent isopeptide bond between Gly76 of the ubiquitin and the ε-amino group of a lysine residue on the substrate [4]. Indeed, a ubiquitin deletion mutant lacking terminal Gly residues (ΔGG) cannot be conjugated to substrate proteins. Hence, a ΔGG ubiquitin mutant is often used as a negative control for in vitro ubiquitination reactions in biochemical studies of E3 ubiquitin ligases.

## 2. Types of Ubiquitination

**Figure 2:**
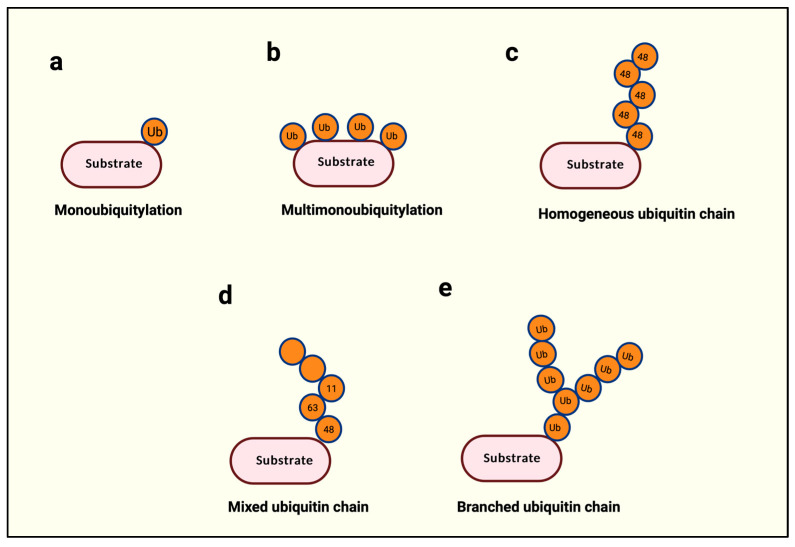
Types of ubiquitination. (**a**) A single ubiquitin is attached to the substrate protein. (**b**) Several ubiquitin molecules are attached to multiple lysine residues within the same protein. (**c**) The same type of ubiquitination is used throughout a single polyubiquitin chain. (**d**) More than two different types of ubiquitin linkages are used in a polyubiquitin chain. (**e**) A single ubiquitin is used for two or more ubiquitin attachments subsequently. Created with BioRender.com.

The addition of a single ubiquitin molecule to a protein substrate is called monoubiquitination (Figure 2a). Monoubiquitination is non-degradative, enhances protein–protein interactions, changes protein activity, and controls subcellular localization [4]. It could specifically occur on a single lysine, such as Lys164 in proliferating nuclear antigen (PCNA) and Lys519 in SMAD4 proteins, or it might be domain-specific such that multiple lysine residues are ubiquitinated with a single ubiquitin each, as in epidermal growth factor receptor (EGFR) (Figure 2b) [4].

Within ubiquitin, the N-terminal methionine (Met-1) and seven internal lysines (K6, K11, K27, K29, K33, K48, and K63) serve as acceptors for the successive addition of a ubiquitin, leading up to the building of a ubiquitin chain with a possibility of eight distinct types of ubiquitin linkages [4]. The building of such a ubiquitin chain is called polyubiquitination. The length of a polyubi-chain may vary from as little as two successively linked ubiquitin proteins to 10 or more. Polyubi-chains can be homogenous if the chain is built with a single linkage type, as in Met-1-, K11-, or K48-only linkages (Figure 2c), or heterogeneous if different linkages alternate in successive positions on a newly added ubiquitin (Figure 2d) [4]. Finally, if a single ubiquitin is modified with multiple ubiquitin molecules, this phenomenon is called branched ubiquitination (Figure 2e) [4].

## 3. Why Do Cells Need Ubiquitination?

Each type of ubiquitination serves as a signal [4]. It is a cellular signal that helps the cell decide the fate of the modified protein. For example, K48-linked polyubiquitin chains target proteins for proteasomal degradation, while K11 and K48 branching of chains enhance proteasomal degradation [5]. Thus, a K11/K48-linked ubiquitin-conjugated protein is readily recognized by the 26S proteasome, a large protease complex that degrades the tagged protein into small peptides. This process is essential for regulating protein levels within the cell, ensuring that damaged, misfolded, or no longer needed proteins are efficiently removed.

Similarly, K6- and K63-linked chains are required for proteasome-independent cellular functions, including protein–protein interactions during DNA damage response, while K63/M1 and K63/K48 branched ubiquitin chains may regulate immune response [5,6,7]. In a resting cell, most polyubiquitination is K48 linked, followed by K63 linkage [5]. The other linkages are mostly context dependent, so less is known about their physiological role. Nevertheless, substrates linked with K11/K48-branched ubiquitination are rapidly turned over during cell cycle transitions [5], while K6- and K33-linked chains are abundant during DNA damage response [8]. Thus, the cell uses diverse ubiquitin linkages as a signal for protein fate decisions.

## 4. The UPS and Its Role in Human Cancers

The UPS regulates a wide variety of biological processes. During cell cycle progression, some key regulatory proteins like cyclins and cyclin-dependent kinase inhibitors are promptly degraded, ensuring proper progression through the cell cycle phases. The UPS members play a critical role in the DNA damage response and DNA repair, as proteins involved in sensing and repairing DNA damage are tightly regulated by ubiquitination-mediated proteasomal degradation. Additionally, the UPS controls the degradation of antigenic proteins into peptides that are presented by major histocompatibility complex (MHC) class I molecules on the cell surface, playing a key role in immune surveillance [9,10]. Thus, dysregulation of the UPS genes could directly or indirectly contribute to human cancers. For example, the aberrant degradation of tumor suppressors like p53 or the stabilization of oncoproteins due to faulty ubiquitination can drive uncontrolled cell growth [11].

Cancer cell metastasis is another aspect of cancer biology actively controlled by several E2 and E3 ubiquitin ligases. E2 enzymes can contribute to building ubiquitin chains crucial for activating pathways like NF-κB and TGF-β signaling, which are crucial in cancer cell survival, inflammation, and migration [12]. Since these pathways are associated with inflammation and metastasis, overexpression of several E2s is known to be associated with cell proliferation, migration, and metastasis in multiple human cancers, including breast, pancreatic, colorectal cancer (CRC), prostate, and lung cancers [13].

Below, we have summarized the role of the most notable UPS members in physiology and cancers.

## 5. Ubiquitin Activating Enzyme (E1)

UBA1 is the first enzyme in the ubiquitination cascade. About eight E1 conjugating enzymes are found in mammalian cells, but only two are specific for ubiquitination, UBA1 and UBA6 (also known as UBE1L2) [14]. E1 enzymes are unique in the ubiquitination cascade because they are the only enzymes that require ATP for ubiquitin activation. This property can be therapeutically exploited, as will be seen in the later example. E1 enzymes are essential for the ubiquitination process that begins with the activation of ubiquitin by the E1 enzyme in an ATP-dependent manner. The E1 enzyme catalyzes the formation of a high-energy thioester bond between the C-terminal glycine of ubiquitin and a Cys residue on the E1 enzyme itself. This process can be broken down into the following four steps:

1. Ubiquitin Binding: The E1 enzyme binds to free ubiquitin molecules via its ubiquitin-binding domain.
2. Adenylation: In the presence of ATP, the C-terminal glycine residue of ubiquitin is adenylated, forming a ubiquitin-adenylate intermediate.
3. Thioester Bond Formation: The E1s contain a “Cys” domain—which attacks the acyl-adenylated ubiquitin and forms a thioester linkage between the ubiquitin and the catalytic cysteine. This reaction results in the formation of an E1–ubiquitin complex.
4. Transfer to E2 Enzyme: The activated ubiquitin is subsequently transferred from the E1 enzyme to an E2 ubiquitin-conjugating enzyme, which, in conjunction with an E3 ubiquitin ligase, ultimately transfers ubiquitin to the target protein [4].

## 6. UBA1 and Cancer

E1 controls cell cycle regulation, apoptosis, and DNA damage response [15,16]. Particularly, monoubiquitination of histone proteins is a hallmark of DNA damage response, and E1, in collaboration with E3 ubiquitin ligases like BRCA1 and a RING domain protein (BARD1), contributes to efficient DNA damage repair [17]. Dysregulation of the E1 enzyme can lead to defective DNA repair mechanisms [15]. This can increase the likelihood of mutations and chromosomal aberrations, contributing to cancer development.

Although examples of UBA1 driver mutations in solid cancers are rare, more recently, at least three somatic mutations that alter the start codon of UBA1 in hematopoietic precursor cells have been reported [18]. These mutations cause loss of UBA1 activity and result in the expression of an impaired non-catalytic form of this enzyme. The patients develop an autoimmune disease called VEXAS syndrome with hallmarks of autoimmunity, including fever, cytopenia, dysplastic bone marrow, and pulmonary inflammation. Somatic mutations in hematopoietic stem cells may lead to myeloid cancers and bone marrow failure syndromes. Indeed, in another study, Maki et al. found novel variants of *UBA1* in patients from a cohort of hematological malignancies with a family history of lung cancer [19]. Finally, *UBA1* splice variants have recently been detected specifically in CRC [20]. However, the clinical significance of these alternate splicing is not known.

## 7. Ubiquitin-Conjugating Enzymes (UBE2)

E2 ubiquitin-conjugating enzymes have a highly conserved core domain of around 150 amino acids called the ubiquitin-conjugating (UBC) domain. This domain includes a Cys residue that forms a thioester bond with ubiquitin, enabling the transfer of ubiquitin to the target protein. E2 enzymes are typically categorized based on their sequence homology and domain structure. E2 enzymes are highly conserved across species, and almost all E2s contain a characteristic His-Pro-Asn (HPA) peptide residue in their UBC domain [12]. Depending on the positioning of the HPA peptide in the UBC domain, E2s are broadly characterized into four classes (Class 1–4) [12]. Class I contains only the catalytic UBC (ubiquitin-conjugating) domain. Class II has an additional N-terminal extension besides the catalytic UBC domain. Class III has a C-terminal extension beyond the catalytic UBC domain. Finally, class IV contains N-terminal and C-terminal extensions flanking the UBC domain. These extensions affect the activity and substrate recognition and facilitate potential interactions with E3 ligases and substrates [12].

E2 enzymes work together with E1 and E3 ligases to carry out substrate-specific ubiquitin ligation. There are approximately 40 known E2 enzymes in the mammalian genome. A single E2 can work with multiple E3 ligases, and this pairing often depends on the type of E3 ligase, the cellular context, and the type of required ubiquitin linkage. Therefore, E2 enzymes are believed to determine the topology of ubiquitin linkage types [4].

Typically, the E2 enzyme’s core functions include:

1. Ubiquitin Transfer: Receive an activated ubiquitin from the E1 enzyme and form a temporary bond with the ubiquitin.
2. Substrate Specificity: The structure and interaction of the E2–E3 complex determine the substrate specificity and the type of ubiquitin chain formed.
3. Ubiquitin Chain Formation: Determine the type of ubiquitin chain on the substrate. Different types of polyubiquitin chains are formed depending on the specific E2 and E3 combinations involved.

E2 enzymes, in partnership with E3 ligases, regulate the timely degradation of cell cycle regulators such as cyclins and cyclin-dependent kinase inhibitors, ensuring proper cell cycle progression [21]. E2 enzymes play a critical role in the ubiquitination of proteins involved in DNA damage recognition and repair, facilitating the recruitment of repair machinery to damaged sites [12]. E2 enzymes are also involved in ubiquitin-mediated signaling pathways, regulating the stability and activity of key signaling molecules and influencing pathways such as NF-κB, WNT, and TGF-β [22,23,24]. E2 enzymes help maintain the balance between cell survival and cell death by controlling the degradation of pro-apoptotic and anti-apoptotic proteins [25].

## 8. UBE2s and Cancer

The precise regulation of protein ubiquitination is crucial for maintaining cellular homeostasis. Dysregulation of E2 enzymes, either through overexpression, mutation, or altered activity, can lead to aberrant protein ubiquitination, contributing to the initiation and progression of cancer. The role of E2 enzymes in cancer can be explored through various mechanisms, including the deregulation of cell cycle control, disruption of DNA repair mechanisms, resistance to apoptosis, and aberrant signaling.

Some of the most widely studied E2 enzymes and their proposed action in the pathophysiology of cancer are given below:

UBE2C regulates mitosis by mediating ubiquitination and degradation of cell cycle regulators in mammalian cells. It is frequently overexpressed in cancers such as breast, lung, and prostate cancers. UBE2C overexpression is associated with poor prognosis, leading to unchecked cell cycle progression and promoting tumorigenesis [26]. Recent work demonstrated that the UBE2C/APC^CDH1^ ubiquitination-mediated degradation of DEPTOR promotes lung cancer by increasing the mTOR signaling [27].

UBE2S is typically involved in cell cycle progression via its ability to perform K11-specific linkage of proteins needed to be rapidly turned over for the cells to move through the cell cycle [5]. UBE2S might be a prognostic marker of tumor suppressor 53 (TP53) activity in hepatocellular carcinoma as it is partly responsible for TP53 proteasomal degradation [26]. Additionally, short hairpin-mediated inhibition of *UBE2S* in melanoma cells leads to rapid cell cycle arrest [26]. Conversely, UBE2S overexpression destabilizes cell cycle inhibitors and contributes to cancer cell proliferation [13]. Dysregulation of UBE2S can lead to the accumulation of β-catenin, activating WNT target genes and promoting tumorigenesis in cancers such as CRC [28].

UBE2T is involved in the Fanconi anemia (FA) DNA repair pathway and is often overexpressed in cancers such as breast and gastric cancers. Overexpression of UBE2T enhances the ubiquitination and degradation of FANCD2, a key protein in the FA pathway, leading to impaired DNA repair and increased genomic instability [29,30]. Genetic inhibition of UBE2T in bladder and gastric cancer cells blocks cell proliferation and colony growth formation, underscoring the importance of this E2 in cancer cell growth and proliferation [31,32].

UBE2D is another E2 family (UBE2D1, UBE2D2, UBE2D3, and UBE2D4) with distinct isoforms roughly sharing 88% sequence homology. This E2 is associated with TP53 protein ubiquitination-mediated degradation, and its overexpression is also associated with epithelial-to-mesenchymal transition (EMT)—a process required for primary cancer cells to metastasize and colonize distant organs [26,33]. Overexpression of UBE2D enzymes can enhance the degradation of TP53, leading to reduced apoptosis and increased survival of cancer cells [34]. UBE2D1 has been shown to interact with MDM2, an E3 ligase that targets TP53 for degradation 1 [35]. Dysregulation of UBE2D activity can influence the stability of TP53 and other apoptotic regulators, contributing to cancer development. Hence, UBE2D targeting may be an interesting therapeutic strategy in restoring the TP53 protein function to block cancer.

Other E2s, such as UBE2L3, also known as UBCH7, and UBE2A, are involved in NF-κB and WNT signaling pathways. UBE2L3, which largely couples with the HECT family of E3 ubiquitin ligases, is involved in the ubiquitination of proteins in the NF-κB pathway [36]. NF-κB is a transcription factor that promotes gene expression in inflammation, cell survival, and proliferation. Dysregulation of UBE2L3 can lead to the aberrant activation of NF-κB signaling, contributing to inflammation and cancer development [36]. Whereas UBE2A has been shown to regulate the stability of β-catenin, a key mediator of WNT signaling [37].

## 9. E3 Ubiquitin Ligases

The UPS is centered around the E3 ubiquitin ligases, which are enzymes responsible for attaching ubiquitin to substrate proteins. This “tagging”, although not only restricted to, largely marks the proteins for degradation by the proteasome, a crucial process for maintaining cellular function and integrity. Each E3 ligase targets a specific set of substrates, making them critical regulators of cellular processes. Various factors, including the presence of specific recognition motifs in the substrate, post-translational modifications, and interactions with adaptor proteins, determine the specificity of E3 ligases. Dysregulation of E3 ligases has been linked to cancer; they can either suppress tumor formation by breaking down oncogenic proteins or promote cancer by targeting tumor suppressors for degradation. This section examines the role of E3 ubiquitin ligases in cancer, delving into their mechanisms, the effects of their dysregulation, and their potential as therapeutic targets.

The mammalian genome encodes only a few E1 and E2 enzymes but at least 800 E3 ligases, meaning that E1 and E2 enzymes must cooperate with multiple E3 ubiquitin ligases to target a wide range of protein substrates for ubiquitination. E3 ubiquitin ligases control nearly every aspect of cellular life, from the cell cycle and DNA damage response to protein turnover, protein secretion, inflammation, and immunity [38]. As a result, numerous E3 ligases are frequently mutated or deregulated in human diseases, including cancer. This makes them a particularly interesting therapeutic target within the UPS. While interest in E3 ubiquitin ligase studies has increased over the past few decades, many E3 ligases still have unknown functional biology.

E3 ligases distinguish from each other based on the mechanism of how a substrate receives a ubiquitin from the E2-conjugating enzyme. Typically, based on their mechanism of action and protein structure, the E3 ubiquitin ligases are categorized into the following three types:

## 10. HECT (Homologous to E6-Associated Protein C-Terminus) E3 Ligases

The HECT family of ligases contains ~30 E3 ligases characterized by a conserved HECT domain, whereas a more variable N-terminus is often required for interaction with E2 enzymes and governs substrate specificity. The HECT domain is approximately 350 amino acids long and is responsible for the enzyme’s catalytic activity. It contains the active Cys, and the ubiquitin is transferred from the E2 to this active Cys residue, which forms a thioester bond with ubiquitin before transferring it to the substrate. This two-step process gives HECT ligases more control over the ubiquitination process, allowing them to regulate the length and topology of the ubiquitin chain before being conjugated to the substrate. The HECT E3 ligases are involved in diverse cellular functions, including protein trafficking, immune response, and cell proliferation. Recently, HECT E3 ligases have been shown to branch ubiquitylate proteins during NF-κB pathway activation, proteotoxic stress, and apoptosis [6,39,40]. Thus, HECT-E3 ligases emerge as branching enzymes in the protein ubiquitination cascade.

HECT E3 ligases are further divided into subfamilies based on their N-terminal domains, determining their substrate specificity and interaction with other regulatory proteins. Some of the major subfamilies of HECT E3 ligases include NEDD4 (neural precursor cell expressed developmentally down-regulated protein 4), HERC (HECT and RCC1-like domain-containing protein), and other individual HECT E3 ligases such as E6AP, HUWE1, UBR5, and TRIP12 [41].

## 11. HECT Ligases in Cancer

The NEDD4 subfamily of HECT E3 ligases is extensively studied in cancer. Members of this subfamily, including NEDD4-1, NEDD4-2, and ITCH, are involved in regulating oncogenic and tumor suppressor proteins. Specifically, NEDD4-1 targets the tumor suppressor PTEN for degradation, activating the PI3K/AKT signaling pathway, which promotes cell survival and proliferation [42,43] (Table 1). Overexpression of NEDD4-1 has been observed in breast, prostate, and lung cancers [44,45]. NEDD4-2, or NEDD4L, regulates the degradation of substrates like the epithelial sodium channel (ENaC) and SMAD2/3, impacting TGF-β signaling [46]. NEDD4-2 also targets cytoplasmic YAP1 to regulate autophagic cell death in breast cancer cells [47]. Dysregulation of NEDD4-2 is associated with pulmonary fibrosis [48] and cancer progression, particularly lung and CRC, affecting cell proliferation and metastasis [43,44,45,49,50].

ITCH plays a crucial role in apoptosis and immune responses. ITCH targets pro-apoptotic proteins like p73 for degradation, promoting cell survival [51]. Under conditions of extracellular stress, c-Jun turnover is enhanced by JNK phosphorylation and ubiquitination by ITCH [52] (Table 1). Dysregulation of ITCH activity has been linked to apoptosis evasion in cancer cells, contributing to tumor growth and therapy resistance [53]. USP9X, a deubiquitinase for ITCH, is focally repressed in pancreatic cancer (PDAC), leading to reduced ITCH protein levels and aggressive PDAC phenotypes [54].

The HUWE1 gene encodes for a large HECT-domain E3 ubiquitin ligase and is mutated in several human cancers, including ~15% of CRC [55]. HUWE1 is involved in diverse cellular processes. For example, HUWE1 suppresses tumor formation in the mammalian gut by directly targeting β-catenin—the major WNT pathway activator [56]. Additionally, HUWE1 targets proto-oncogenic proteins, including c-MYC and MCL1, for proteasomal degradation and blocks tumorigenesis [41,57] (Table 1). Indeed, mice with intestine-specific *Huwe1* deletion show aggressive intestinal tumors and increased protein levels of MYC [55].

*UBR5* is often amplified in human cancers. The true extent of UBR5 function in mammalian biology is not well established. However, as it is frequently amplified in many human cancers, this suggests that it acts as an oncogene. In agreement with this, UBR5 is shown to enhance WNT signaling by targeting negative regulators of the WNT pathway, Groucho/TLE [58] (Table 1).

Another HECT E3 ligase, TRIP12, in collaboration with UBR5, protects mammalian cells from genomic instability by maintaining physiological levels of E3 ubiquitin ligase RNF168 [59] (Table 1). Interestingly, we recently discovered that TRIP12 might act as a branch ubiquitylating enzyme for another tumor suppressor protein, FBW7, to enhance its proteasomal degradation, thereby leading to chemotherapy resistance [60] (Table 1). Others have found TRIP12-branched ubiquitination in the proteolysis-targeting chimera (PROTAC)-mediated targeted protein degradation [61]. TRIP12 also regulates the mammalian cell cycle in multiple cell lines [62,63] and has been recently shown by our lab to be an evolutionarily conserved modulator of TGF-β signaling [64]. TRIP12 is also involved in acinar-to-ductal metaplasia, which is an initiating event in PDAC. Genetic inhibition of *Trip12* in PDAC mouse models blocks the progression of PDAC and PDAC-associated metastasis [65].

SMURF1 and SMURF2 are HECT E3 ligases that regulate TGF-β signaling by targeting SMAD proteins for ubiquitination and degradation [66,67]. TGF-β signaling has a dual role in cancer, acting as a tumor suppressor in early stages and promoting metastasis in later stages. Dysregulation of SMURF1 and SMURF2 has been associated with cancer progression, especially in breast and lung cancers, where they contribute to EMT and metastasis [68]. Therefore, SMURF1 and SMURF2 may promote tumorigenesis by modulating and regulating the TGF-β signaling pathway (Table 1).

The HERC subfamily of HECT E3 ligases includes HERC2 and HERC1. HERC2 is involved in regulating DNA damage repair proteins like BRCA1 and TP53. It interacts with and adds ubiquitin to TP53, which leads to TP53’s degradation and inhibits its ability to suppress tumors [69]. Mutations or dysregulation of HERC2 have been linked to an increased risk of certain cancers, especially breast and ovarian cancers [70]. On the other hand, HERC1 regulates cell proliferation and movement by interacting with the RAS signaling pathway [71]. Dysregulation of HERC family members has been associated with the development of breast, colon, and other cancers, where it may contribute to the abnormal activation of RAS signaling, promoting tumor growth and metastasis [70].

E6AP, also known as UBE3A, is a HECT E3 ligase that has gained attention for its role in human papillomavirus (HPV)-associated cancers. E6AP degrades the tumor suppressor TP53 when the HPV E6 oncoprotein is present [72] (Table 1). This breakdown prevents TP53-mediated apoptosis and helps HPV-infected cells survive. This process is especially important in cervical cancer and other HPV-related cancers. In addition, E6AP has been linked to other types of cancers by regulating the stability of the proteins that control the cell cycle, DNA damage repair, and signal transduction [73]. In breast cancer, E6AP affects the stability of estrogen receptor α (ERα) and contributes to the hormone-dependent growth of breast cancer cells [74].

## 12. RBR E3 Ligases [RING-IBR (In-Between-RINGs)]

With ~100 members, the RBR E3 ubiquitin ligase family is the second largest class of E3 ubiquitin ligases. They are characterized by the presence of three typical domains, including two RING fingers (RING1 and RING2) and a central IBR zinc-binding domain [75]. The RBR E3 ligases are distinct in the sense that they possess the hybrid mechanistic feature of the RING (to be explained later) and HECT-type E3 ligases. First, the E2~ubiquitin intermediate interacts with the RING1 domain of RBR. Second, the ubiquitin molecule from the E2 is transferred to the active Cys of the RING2 domain and finally conjugated on the substrate protein [75]. RBR ligases are also inducted in many cellular pathways. The most noticeable example is the linear ubiquitin chain assembly complex (LUBAC). LUBAC comprises SHARPIN, HOIP, and HOIL, forming a ~600 Kd functional ternary complex. LUBAC is unique in the sense that, unlike typical E3 ubiquitin ligases, LUBAC catalyzes the formation of an amide bond between Gly76 of ubiquitin and the α-NH2 group of the first methionine of another ubiquitin, thus giving rise to Met1-type of ubiquitin linkage called linear polyubiquitination [75]. The LUBAC controls inflammation and immunity by engaging in the NF-κB pathway—a major transcription factor activated by inflammatory signals, and its components are essential for life [9,10,76].

## 13. RBR E3 Ligases in Cancer

LUBAC-mediated NF-κB activation leads to inhibition of cell death, enhances inflammation, and promotes tumorigenesis [9,10,75]. LUBAC components are involved in the chemoresistance of CRC and lung cancer cells [77,78]. Hypoxia-inducible factor (HIF)-induced HOIL1 expression results in polyubiquitination and degradation of PKCζ, which promotes tumorigenesis in lung cancer [79]. On the contrary, silencing LUBAC components reduces tumor size in lung cancer and sensitizes them to chemotherapy [77]. The LUBAC complex also plays a role in regulating apoptosis through the ubiquitination of components of the apoptotic machinery [80,81,82]. Dysregulation of LUBAC function can lead to the evasion of apoptosis, a hallmark of cancer, and contribute to tumor cell survival and resistance to therapy [77]. Additionally, LUBAC controls breast cancer development, and its components might be overexpressed in breast cancer compared to adjacent normal tissue [83]. Thus, the LUBAC complex might be a potential therapeutic target in certain cancers [84].

Parkinson protein 2 (PARK2, also known as Parkin) is another well-known RBR E3 ligase. It has a vital role in maintaining mitochondrial quality control and mitophagy, and it is a risk gene in Parkinson’s disease since its mutations are strongly associated with Parkinson’s disease [85]. Interestingly, the pan-cancer genetic analysis showed that the PARK2 gene is frequently deleted in human cancers [86]. In agreement with that, recent studies show that PARK2 acts as a tumor suppressor. PARK2 suppresses breast cancer metastasis by ubiquitinating and degrading HIF-1α [87]. Therefore, if PARK2 is mutated, HIF-1α starts to accumulate and promotes breast cancer metastasis [87]. Moreover, Parkin deficiency promotes cyclinE accumulation, leading to G1/S cell cycle progression and uncontrolled cell proliferation [86]. Additionally, its mutation activates PI3K/AKT signaling in cancer cells by inactivating PTEN, a tumor suppressor, through S-nitrosylation and ubiquitination [88]. PI3K/AKT activation promotes metabolic reprogramming—a hallmark of cancer.

The Ariadne family of RBR E3 ligases consists of ARIH1 and ARIH2, which are vital in regulating protein degradation in DNA damage response and immune signaling. ARIH1 is responsible for the ubiquitination and breakdown of proteins, including EIF4E2 and SUN2, associated with the DNA damage response and cell cycle regulation [89,90]. Dysregulation of ARIH1 has been associated with the development of cancer, particularly breast cancer and gastric cancer, where it affects the stability of key regulatory proteins involved in tumor suppression and DNA repair [91,92]. ARIH2 also contributes to regulating immune responses by modulating the ubiquitination of immune signaling molecules [93]. Dysregulation of ARIH1 (discussed later) has been linked to immune evasion in cancer, where it hampers anti-tumor immune responses and supports tumor growth [94].

## 14. RING (Really Interesting New Gene) E3 Ligases

The RING E3 ubiquitin ligases are characterized by their RING or U-box fold catalytic domain. RING-type E3 ligases interact with E2 enzymes by their RING finger domain and mediate the direct transfer of the ubiquitin from E2 to the target protein [95]. RING E3 ligase family is the biggest class of E3 ligases, with over 600 predicted members in the human genome [95]. One classic family member is SKP/CUL1/F-box (SCF)-type E3 ubiquitin ligases. Unlike HECT-type E3 ligases, this is a multiprotein complex consisting of a substrate adaptor, SKP1 and cullin (CUL) scaffolds, and a RING E3 ligase [95].

The rigid scaffold “CUL” provides stability to the cullin–RING ligase complex. In addition, the critical subunit of the SCF-type cullin–RING ligases is the substrate adaptor, BTB or F-box-containing protein, depending on the cognate cullin in the complex [95]. The F-box proteins, including β-TRCP1/2, SKP2, and FBW7, provide substrate specificity to SCF-type ubiquitin ligases and regulate the cell cycle, proliferation, apoptosis, and DNA damage response [95].

Another multi-component RING-domain-containing ligase is the anaphase-promoting complex/cyclosome (APC/C). Like the SCF family, APC/C has CUL and RING-finger domains [96]. In addition, APC/C is activated by two co-activators: Cdc20 and Cdh1 [96]. APC/C targets cyclin B and securin for proteasomal degradation to initiate sister-chromatid separation and exit from mitosis [96]. APC/C also regulates different cellular processes, including cell cycle regulation, genome stability, apoptosis, autophagy, and tumorigenesis [97].

RNF ligases are another class of RING-type E3 ligases that play a role in various cellular functions with ~250 members. The RING finger domain in RNFs typically consists of a series of conserved cysteine and histidine residues coordinating two zinc ions [98]. This domain helps transfer ubiquitin from an E2 ubiquitin-conjugating enzyme to a specific substrate. Unlike HECT and RBR E3 ligases, which form a ubiquitin–E3 intermediate before transferring ubiquitin to the substrate, RING-type E3 ligases act as scaffolds and directly catalyze the transfer of ubiquitin from the E2 enzyme to the substrate [98]. RNF E3 ligases can function as monomers, homodimers, or as part of larger multi-subunit complexes. Their activity and substrate specificity are often regulated by additional domains or through interactions with other proteins. The diversity of the RNF family allows for a wide range of substrates and functions, making them essential regulators of cellular homeostasis [98].

TRIM proteins are another class of RING E3 ligases containing three distinct domains. Like RNF E3 ligases, the TRIM family contains the RING finger domain, a specialized zinc-binding domain that mediates the transfer of ubiquitin from an E2 ubiquitin-conjugating enzyme to the substrate [99]. In addition, a coiled-coil domain facilitates homo- or hetero-dimerization of TRIM proteins, which is often necessary for their activity [99]. TRIM E3 ligases are involved in multiple cellular pathways, including apoptosis, protein trafficking, cellular differentiation, and immune response [100,101].

## 15. RING E3 Ligases in Cancer

The SCF-(FBW7) member of the RING family targets oncogenic proteins such as c-MYC, Notch1, CyclinE, and c-JUN for proteasomal degradation [27]. Loss-of-function *FBW7* mutations drive T-cell acute lymphoblastic leukemia (T-ALL) partly because FBW7 targets Notch1 for proteasomal degradation in the hematopoietic system. This keeps the levels of oncogenic Notch proteins in check and prevents the transformation of normal to cancer cells [102] (Table 2). In addition, MCL-1, which is an anti-apoptotic (BCL2 family) protein and an FBW7 substrate, is frequently amplified or stabilized due to loss-of-function *FBW7* mutations in cancer [103]. This increase in MCL-1 abundance is strongly associated with chemotherapy resistance and poor clinical outcome. Indeed, genetic targeting of MCL-1 in preclinical models has validated MCL-1 as a promising therapeutic target [104]. Other cancers notable for frequent *FBW7* mutations are CRC, lung, uterine, and pancreatic cancer. Several pathways have been identified that are reported to converge on FBW7 activity, including the deubiquitinase USP9X, E3 ligase TRIP12, dual specificity kinase DYRK2, and peptidylprolyl cis/trans isomerase (PIN1) [60,105,106,107]. Perturbations of these proteins may affect FBW7 function and ultimately aggravate cancer and chemotherapy resistance [60,105,106,107].

SKP2, another substrate adaptor of the CUL-RING ligase family, targets tumor suppressors p21 and p27 for proteasomal degradation in cancer [108]. Additionally, SKP2 regulates the proteasomal degradation of oncogenic protein c-MYC and its transcriptional activity [109]. Thus, several lines of evidence suggest that the SKP2 E3 ligase might promote tumorigenesis in human tissues. Indeed, the *SKP2* gene is frequently mutated in human cancers, but the exact mechanism of SKP2 as an oncogene requires in-depth research. In line with those findings, inhibition of SKP2 in skin cancer cells stabilizes tumor suppressor KLF4 and p21, leading to apoptotic cell death [110]. Several other studies demonstrate the validity of targeting SKP2 in other forms of cancer, including leukemia [111,112,113,114]. CUL4A, part of the SKP2-CRL4 E3 ligase complex, also targets tumor suppressors for proteasomal degradation, including p21^Cip1^ and p27^Kip1^ [115].

Much like FBW7 and SKP2, β-TRCP1/2 is an important regulator of the cell cycle G1-to-S transition [116]. CDC25A protein is a phosphatase that activates CDC2 during the G1 phase of the cell cycle [117]. For the cells to progress to the S phase, CDC25a levels must be reduced. β-TRCP1/2 binds to CDC25a in a phosphorylation manner and tags it with polyubiquitination for proteasomal degradation [116]. Additionally, it targets β-catenin, a key effector of the WNT pathway, for degradation [118]. Mutations in the *APC* gene, which regulates β-TRCP-mediated degradation of β-catenin, lead to the accumulation of β-catenin and activation of WNT target genes, driving CRC progression [119]. Additionally, non-degradative ubiquitination of oncogenic protein c-MYC by β-TRCP1 prevents its FBW7-mediated proteasomal degradation [120]. Thus, β-TRCP1/2 may act as a tumor suppressor or an oncogenic protein, depending on the context.

VHL is an E3 ligase that targets hypoxia-inducible factor (HIF) for degradation under normoxic conditions [121]. Loss of VHL function leads to the accumulation of HIF and the activation of hypoxia-responsive genes, contributing to tumor angiogenesis and cancer progression [121,122]. VHL also binds to and inhibits the cellular levels of Akt. Cancer-associated Akt mutations result in loss of VHL/Akt interaction, thereby allowing cancer cells to proliferate even under hypoxic settings [123]. Thus, VHL is a tumor suppressor E3 ligase (Table 2).

SPOP is a substrate recognition component of CUL3–RING ligase, which targets the BET proteins for proteasomal degradation. BET proteins bolster the functions of key oncogenes by increasing the expression of these drivers, such as c-MYC in leukemia [124,125], or by enhancing the transcriptional activities of oncogenic factors, such as Androgen receptor (AR) and Transcription regulator ERG in prostate cancer [126]. *SPOP* is frequently mutated in prostate cancer. Mutations in the *SPOP* gene result in the loss of its activity towards the BET proteins and, ultimately, the development of resistance against BET inhibitors [127].

*SPOP* mutations also promote p62/SQSTM1-dependent autophagy and NRF2 activation in prostate cancer [128,129] (Table 2). Thus, *SPOP* mutational status may serve as a prognostic marker for treating prostate cancer patients in a precision medicine manner.

Another BTB domain-containing protein gene, *KEAP1*, is frequently mutated in lung cancers [130]. KEAP1 is an E3 ligase that targets NRF2 for degradation. NRF2 is a transcription factor involved in oxidative stress response (Table 2). Mutations in *KEAP1* stabilize NRF2, promoting cancer cell survival and resistance to chemotherapy [130]. Indeed, *Keap1* deletion enhanced lung tumor growth in mouse models [131].

BRCA1, a well-established tumor suppressor, is also an E3 ubiquitin ligase involved in DNA damage repair. Mutations in *BRCA1* lead to defects in homologous recombination, increasing the risk of breast and ovarian cancers [17,132] (Table 2). BRCA1 also regulates the degradation of ERα, a key driver of hormone-responsive breast cancers [133].

Several RNF ligases have been shown to have distinct roles in human cancers. RNF43 is a tumor suppressor E3 ligase that negatively regulates WNT signaling by promoting the degradation of WNT receptors [134]. Loss-of-function mutations in *RNF43* are common in CRC and gastric cancer, leading to hyperactivation of WNT signaling and tumorigenesis [135]. In addition, *RNF43* is frequently mutated in mucinous ovarian carcinomas [136]. Interestingly, *RNF43* mutations in pancreatic cancer shift the pancreatic cancer dependency to WNT signaling rather than oncogenic KRAS [137], further underscoring the importance of this E3 ligase as a negative regulator of WNT signaling and a tumor suppressor.

RNF203 is another cell-surface E3 ubiquitin ligase frequently altered in adrenocortical carcinoma [138]. RNF167 targets CASTOR1 for proteasomal degradation and activates mTOR signaling to promote cancer progression [139,140].

RNF5 is another membrane-bound RING ligase that is involved in antiviral response by targeting the STING protein with Lys48-linked polyubiquitination for proteasomal degradation [141]. Recent work has implicated the overexpression of *RNF5* in acute myeloid leukemia (AML). High *RNF5* expression is associated with poor AML prognosis, and inhibition of RNF5 decreases AML growth and sensitizes it to histone deacetylase (HDAC) inhibitors in preclinical models [142]. Thus, RNF5 might be a potential drug target in AML.

RNF8 and RNF168 are the guardians of genomic integrity by ubiquitylating histone proteins, and several substrates involved in DNA damage repair pathways [59,143] (Table 2). A recent work suggested the role of RNF8 in breast cancer via negative regulation of Notch proteins [144]. RNF168 has also been shown to regulate the R-loop resolution in BRCA1/2 deficient tumors [145].

Recent work demonstrated a tumor suppressor role for RN128 in the colitis-mediated CRC by targeting IL6 receptors and reducing colonic inflammation [146], and RNF61 is shown to ubiquitinate and enhance SNIP1 ubiquitination-mediated degradation to promote cancer metastasis by activating the TGF-β signaling [147]. On the contrary, RNF63 is proposed as a tumor suppressor in non-small cell lung cancer by ubiquitylating a proto-oncogene, PABPC1 [148].

TRIM25 is best known for its role in the innate immune response, where it ubiquitinates the RNA sensor RIG-I, promoting the production of type I interferons [101,149,150]. However, TRIM25 also has oncogenic functions. It is an E3 ligase that ubiquitinates proteins that are involved in EMT and cancer metastasis [151]. TRIM25 is an ERα-responsive gene [152]. It promotes breast cancer cell survival by ubiquitylating and degrading the tumor suppressor ABTF1 [153]. Thus, overexpression of TRIM25 is associated with poor prognosis in breast cancer patients [154].

TRIM28 (KAP1/TIF1β) is a transcriptional co-repressor that interacts with KRAB-domain zinc finger proteins to repress gene expression [155]. It is also a scaffold protein that recruits chromatin remodeling complexes to target genes [156,157]. TRIM28 can modulate TP53 activity by promoting the sumoylation and transcriptional repression of TP53 target genes [158]. *TRIM28* is overexpressed in several cancers, including breast, lung, and CRC [159]. Its role in repressing TP53 activity and altering chromatin structure suggests that TRIM28 promotes oncogenesis by silencing tumor suppressor genes and maintaining an undifferentiated, proliferative state in cancer cells [159]. More recently, KAP1 has been suggested to be responsible for resistance against anti-PD-1 in non-small cell lung cancer (discussed later) [160].

TRIM32 ubiquitinates various substrates, including the TP53 and the c-MYC [161,162]. It has also been implicated in regulating cell proliferation, differentiation, and apoptosis by regulating several cellular pathways. TRIM32 promotes proliferation and chemoresistance by activating the pro-inflammatory NF-κB activity in breast cancer cells [163]. It promotes the proliferation of gastric cancer cells by rewiring the metabolism via activation of the AKT pathway [164]. Overexpression of TRIM32 is associated with poor clinical outcomes in AML [165], and TRIM32 inhibition sensitizes glioma cells to chemotherapy by a TP53-independent pathway [166]. These studies strongly suggest that TRIM32 is most likely an oncogene.

Several data lines suggest that TRIM7 may act as either a tumor suppressor or an oncogene, depending on the context. TRIM7 was first identified as an E3 ligase for the AP1/c-Jun co-activator RACO-1 and was shown to possess K63-ubiquitin linkage activity for RACO-1 [167]. TRIM7-mediated K63 ubiquitination of RACO-1 induced AP1/c-Jun transcriptional activity, and its overexpression led to enhanced lung tumor burden in mice overexpressing TRIM7 [167]. Recent studies suggest TRIM7 acts as a tumor suppressor. It may target SLC7A11 for ubiquitination-mediated degradation in gastric cancer cells, thereby resulting in ferroptosis of those cells [168]. Additionally, TRIM7 targets NF-κB subunit p65 for proteasomal degradation in lung cancer cells and inhibits their proliferation and metastasis [169], providing more evidence in support of its tumor suppressor role.

TRIM21 was first identified as an inhibitor of intracellular viral infection by targeting virions for proteasomal degradation [170]. More recently, it has been shown to target glucose-6-phosphate (G6P) and block glycolysis in cancer cells [171]. PTEN-null cancer cells can directly act on TRIM21 and inhibit its activity to rewire the cancer metabolism by stabilizing the G6P enzyme [171]. Interestingly, a recent report suggested that TRIM21 can specifically interact with mutant TP53 and thereby prevent its gain of function by mediating its proteasomal degradation [172]. These works underscore the importance of TRIM21 as a potential tumor suppressor.

Other TRIM E3 ligases are relatively less well characterized in human cancers. More recently, several TRIM family members have been inducted into the pathophysiology of cancer. TRIM4 is shown to enhance ERα activity in breast cancer cells by targeting SET proteins for ubiquitination-mediated degradation [173]. In the absence of TRIM4, ER+ breast cancer cells become tamoxifen resistant, and TRIM4 was found to be downregulated in ER+ breast cancer, suggesting a tumor suppressor role for this ligase [173]. On the other hand, TRIM17 has been shown to promote the proliferation of gastric cancer cells via ubiquitination-mediated proteasomal degradation of apoptotic protein BAX [174]. This work also demonstrated that *TRIM17* is transcriptionally upregulated in gastric cancer patients, while BAX expression is inversely related to *TRIM17* in those cohorts [174]. TRIM26 is another TRIM E3 ligase that has been shown to act as a tumor suppressor in hepatocellular carcinoma. It can prevent EMT by ubiquitylating the key EMT transcription factor ZEB1 [175]. Thus, mounting evidence suggests that the TRIM family of E3 ligases actively contributes to the pathophysiology of cancer as an oncogene or a tumor suppressor.

## 16. E3 Ubiquitin Ligases in Cancer Immune Evasion and Surveillance

For the cancer cells to survive and continue to grow within the normal tissue, they must mask themselves and provide a classical “eat me not” signal to the body’s immune system. The programmed death ligand 1 (PD-L1) is the cell surface receptor that protects the host tissue from infection elicited immune response. It is often overexpressed in human cancers and allows the tumor cells to escape immune attack. The UPS is expected to play a role in cancer immune evasion by controlling the stability and activity of the PD-1, PD-L1, or MYC proteins, depending on the context and driver mutations. While treatments targeting immune checkpoints like PD-1 and its ligand PD-L1 have been approved for treating human cancers with lasting clinical benefits, many cancer patients do not respond to these treatments, and the reasons for this are only coming to light.

**Modulation of cancer immune surveillance by oncogenic c-Myc:** The transcription factor MYC regulates self-renewal, differentiation, proliferation, and apoptosis [176] and directly regulates the expression of PD-L1 and CD47 [177]. In addition to being a direct transcriptional target of c-MYC, PD-L1 is also posttranslationally stabilized in cancer [178]. Indeed, the genetic inactivation of *c-MYC* induces proliferative arrest, apoptosis, and regression of tumors in vivo, which is dependent on the host’s immune response [177]. Consistent with this, anti-PD-L1 antibodies are the best available cancer immunotherapy to date, including those for melanoma and non-small cell lung cancers.

Increased MYC activity or *MYC* amplification is widespread in human cancers and is causally associated with tumorigenesis and poor clinical outcomes [176,177]. Hence, the level of MYC proteins is tightly regulated in normal tissues. Many E3 ubiquitin ligases negatively regulate MYC protein, including F-Box-containing proteins SKP2 and FBW7 [179], and HUWE1 [55]. Interestingly, c-MYC ubiquitination by SKP2 not only regulates its proteasomal turnover but also regulates its transcriptional activity. Indeed, a lysine-less c-MYC mutant is defective in inducing MYC target genes despite its ability to interact with its obligate binding partner MAX [120]. Thus, this ubiquitination is required for its transcriptional activity, which is different from the degradative ubiquitination of c-MYC. Whether SKP2 globally regulates c-MYC transcriptional activity or only a subset of genes is not known. The molecular details of the type of ubiquitin chain assembly and topology required for transcriptional activation of c-MYC are also unclear.

**PD-L1 modulation by members of the RING family of E3 ligases:** The abundance of the PD-L1 protein is also regulated directly by at least four different RING E3 ligases, including CUL3^SPOP^, STUB1, FBX22, and β-TRCP [129,180,181,182,183]. CMTM6, a transmembrane protein of previously unknown function, was first discovered to interfere with the proteasomal degradation of PD-L1, allowing the cells to escape tumor immunity. CMTM6 could prevent the ubiquitination of PD-L1 by blocking the binding of STUB1 and PD-L1, thus blocking its proteasomal degradation [181].

CUL3-SPOP E3 ligase, in collaboration with cyclin D-CDK4, also targets PD-L1 for proteasome-mediated degradation [180]. Inhibiting CDK4 and CDK6 in vivo increases PD-L1 protein levels by impeding cyclin D-CDK4-mediated phosphorylation of SPOP and promoting SPOP degradation by the anaphase-promoting complex activator FZR1 [180]. Loss-of-function mutations in *SPOP* compromise ubiquitination-mediated PD-L1 degradation, leading to increased PD-L1 levels and reduced tumor-infiltrating lymphocytes [180]. Combining CDK4/6 inhibitor treatment with anti-PD-1 immunotherapy notably enhances tumor regression and significantly improves overall survival in mouse cancer models [180].

Li et al. demonstrated that PD-L1 is heavily glycosylated in multiple cancer cell lines in response to activated EGFR that induces GSK3β phosphorylation, leading to inhibition of the binding of GSK3β to PD-L1. This inhibition of GSK3β interaction with glycosylated PD-L1 inhibits β-TRCP-mediated PD-L1 ubiquitination and degradation [184]. In another work, inhibition of mTOR signaling in multiple cancer cell lines led to the stabilization of PD-L1 via inhibition of β-TRCP [182].

FBX22 has recently been identified to target PD-L1 in non-small cell lung cancer (NSCLC) [183]. The authors demonstrated that CDK5 inhibition can lead to the upregulation of FBX22, which ultimately targets PD-L1 for proteasome-mediated degradation. Inhibiting CDK5 activity in collaboration with cisplatin enhanced the DNA damage-induced cell death and immunotherapy in preclinical models [183].

Another RING E3 ligase that may regulate cancer immunosurveillance is CBL-b. This E3 ubiquitin ligase is involved in T- and B-cell receptor inhibition [185,186]. Due to its expanding roles in innate and adaptive immunity, inhibiting CBL-b activity has been proposed as a potential target for modulating immune therapies in human diseases, including cancer and autoimmune disorders [187].

**Modulation of cancer immune response by Ariadne family member E3 ligase ARIH1:** ARIH1 appears to ubiquitinate PD-L1 in response to GSKα-mediated phosphorylation of PD-L1 [94]. Wu et. al. highlighted these results in a chemical biology screen [94], where they identified EGFR inhibitors to stimulate ARIH1 activity potentially. A better understanding of this pathway could benefit patients with immune checkpoint inhibitor resistance since ARIH1 overexpression increases cytotoxic T-cell activity against xenograft tumors. In contrast, this effect was completely blocked in immune-deficient mice [94]. The same group demonstrated that cisplatin treatment of 4T1-derived murine breast cancer (TNBC) led to upregulation of ARIH1. This stabilization of ARIH1 led to increased infiltration of tumor-associated cytotoxic T cells, reduced tumor growth, and enhanced anti-PD-L1 response [188]. These studies demonstrate harnessing the power of E3 ubiquitin ligase activities against PD-L1 to enhance anti-tumor immunity in cancer.

**Modulation of cancer immunity by TRIM members of the RING ligase family:** TRIM E3 ligases, including TRIM7, TRIM21, and TRIM32, execute a proper immune response against pathogens [101]. These ligases can control cancer immune response by regulating the activity of the master immune modulator transcription factor NF-κB. For example, ependymin-related protein 1 (EPDR1) overexpression in hepatocellular carcinoma leads to cancer immune suppression [189]. TRIM21 ubiquitinates NF-κB, which leads to its lysosomal degradation and immune suppression. EPDR1 inhibits TRIM21/NF-κB interaction, stabilizes NF-κB-mediated transcriptional upregulation of PD-L1 in hepatocellular carcinoma cells, and inhibits immune response [189].

**Downregulation of MHC class molecules by MARCH E3 ligases:** The presentation of tumor antigens on MHC class I molecules is essential for recognizing cancer cells by CD8+ T cells. Tumor cells can evade immune detection by downregulating MHC class I molecules. Membrane-associated RING-CH (MARCH) ubiquitin ligases control the stability, trafficking, and function of immunoreceptors, including MHC class molecules and costimulatory molecule CD86 [190]. For example, the E3 ligase MARCH1 mediates the ubiquitination and internalization of MHC class I molecules, leading to their degradation and reduced antigen presentation [191]. In addition, MARCH1 downregulation in IL10-activated B-cells increases MHC class II expression, suggesting that MARCH1 negatively regulates MHC class II as well. Thus, MARCH1 alterations in human cancers may affect cancer immune response via modulation of MHC class molecules or antigen presentation pathways [190].

**Linkage-Specific Ubiquitination and Cancer Immune Evasion:** K63-linked ubiquitination is vital in apoptosis and immune regulation, influencing cancer survival pathways and cancer immune evasion. It promotes apoptosis via TNF receptor signaling, with RACK1 enabling K63 ubiquitination of MOAP-1 by recruiting TRAF2 for Bax interaction. K63 ubiquitination also influences T-cell function by enhancing transcription factors like FOXP3 in regulatory T-cells and inhibiting Th9 differentiation and anti-tumor activity through PU.1 [192]. Moreover, K63-linked ubiquitination mediated by TRAF6 ligase is critical in regulating key pathways, including mTOR signaling, cancer cell metastasis, and cell death in various cancers [193,194,195,196]. In breast cancer, TRAF6/USP17 facilitates asparaginyl endopeptidase (AEP) ubiquitination to promote metastasis [197], whereas Uev1A-Ubc13 promotes CXCL1 expression and NF-κB activation in CRC, enhancing metastatic potential.

M1-linked or linear ubiquitin chains are crucial for immune signaling, particularly in activating the NF-κB pathway. In cancer, M1-linked ubiquitination may contribute to tumor growth by protecting cells under hypoxic conditions, promoting drug resistance in response to genotoxic and proteotoxic stress, and sustaining inflammatory signaling. These chains also regulate adaptive and innate immunity, making them significant in inflammatory diseases and cancer treatment strategies [198]. Indeed, LUBAC, the sole ubiquitin ligase complex responsible for M1-linked ubiquitin conjugation, is involved in CRC and lung cancer chemoresistance [77,78].

## 17. Strategies to Target UPS Pathway Members

Because of their role in broader biological functions and in human cancers, several UPS members have been proposed as potential therapeutic targets. E1s are relatively easier to target via pharmacological approaches than E2 and E3 ligases due to the requirement of ATP in their enzymatic activity. However, broadscale inhibition of E1 enzyme activity might give unwanted side effects. On the contrary, upregulation and activating mutations of E2 and E3 enzymes in human cancers make them a better therapeutic target because the inhibition would spare the normal cells. However, similar chemical biology approaches can be used to develop and target E2 and E3 ligase inhibitors, as they both lack ATP binding pockets. Some of the most common approaches that have been exploited to pharmacologically target UPS members in human cancers are given below.

## 18. Pharmacological Targeting of UBA1

Given the crucial role of E1 enzymes in cancer, they have emerged as potential targets for therapeutic intervention. Inhibiting the activity of E1 enzymes can disrupt the ubiquitination process, leading to the accumulation of oncogenic proteins and triggering apoptosis in cancer cells. One such inhibitor called TAK-243 (MLN7243) has shown promise in preclinical trials and is tested in clinical trials [199,200,201,202,203]. Cancer cells treated with TAK-243 rapidly lose ubiquitin conjugates, disrupting signaling events, including cell cycle inhibition, enhanced DNA damage, and proteotoxic effects [199,200,201,202,203].

Targeting the E1 enzyme holds significant promise for cancer therapy but also presents some challenges. One obstacle is achieving specificity, as the E1 enzyme ubiquitylates many proteins. Inhibiting the E1 enzyme could result in the unintended accumulation of non-cancer-related proteins, posing a risk to normal cells. Thus, a careful evaluation of TAK-243’s inhibitory effects on global UPS inhibition is warranted. Additionally, the potential development of resistance to E1 inhibitors is a concern, as cancer cells may acquire mutations that prevent inhibitor binding or upregulate alternative pathways. Overcoming these challenges will require the development of combination therapies targeting multiple components of the ubiquitin–proteasome system or other complementary pathways.

Future research is needed to identify specific E1 enzyme isoforms or splice variants that are selectively expressed in cancer cells. Targeting these isoforms could provide a more selective approach to inhibiting E1 activity in cancer while minimizing off-target effects.

## 19. Pharmacological Targeting of UBE2s

E2 enzymes are crucial in cancer-related processes, making them important targets for potential treatments. Strategies to target E2 enzymes may include small-molecule inhibitors, RNA interference (RNAi), and targeted protein degradation approaches. Small-molecule inhibitors that target the active site or interaction interfaces of E2 enzymes have shown potential in preclinical studies. These inhibitors can stop the transfer of ubiquitin from the E2 enzyme to the substrate, preventing the breakdown of key tumor suppressor proteins involved in cancer.

*NSC697923*: This small-molecule inhibitor targets UBE2N (Ubc13) and selectively inhibits the formation of K63-linked polyubiquitin chains catalyzed by UBC13, but not UBCH5c, by preventing the formation of the UBC13-Ub thioester bond. Treatment with NSC69792 leads to the accumulation of DNA damage and reduced NF-κB signaling in cancer cells, ultimately causing cell death in lymphoma and multiple myeloma (MM) cell lines [204].

*CC0651*: CC0651 inhibits UBE2R1, an E2 enzyme involved in cell cycle regulation. It inhibits the interaction between UBE2R1 and its E3 ligase, stabilizing cell cycle regulators and inducing cell cycle arrest in cancer cells [205]. Another small-molecule inhibitor, DHPO, targets UBCH5c [206] and has shown promise in preclinical models of pancreatic cancer, including cell lines and in vivo xenografts.

The development of small-molecule inhibitors targeting E2 enzymes is being explored in preclinical research. However, further investigation is required before these inhibitors can progress to clinical trials. Currently, there are no inhibitors targeting E2 enzymes in clinical trials. Understanding the interactions between E2 enzymes and E3 ligases is crucial for developing effective therapeutic approaches. Structural studies and high-throughput screening methods can help identify important interaction points and potential drug targets. Identifying biomarkers that can predict the response to E2-targeted therapies for patient stratification and treatment monitoring will also be important. Biomarkers linked to the expression levels, mutations, or activity of E2 enzymes could aid in the development of personalized cancer treatments. Therefore, ongoing research in this area, combined with advancements in drug discovery and biomarker development, holds promise for enhancing cancer treatment and patient outcomes by targeting E2 enzyme activity.

## 20. Pharmacological Targeting of E3 Ubiquitin Ligases

Given the plethora of functions E3 ligases regulate in cancer biology, chemotherapy response, and cancer immune surveillance, these enzymes have emerged as promising therapeutic targets. Small-molecule inhibitors that target the E3 ubiquitin ligase activity prevent the formation of the E3 ligase complex and inhibit the ligase substrate interactions have shown promise in preclinical and clinical studies [207]. One area of intense investigation is identifying and targeting E3 ubiquitin ligases that are directly responsible for ubiquitination-mediated degradation of tumor suppressor proteins. Some of the most prominent approaches for targeting E3 ubiquitin ligases are listed below.

## 21. Inhibitors of HECT E3 Ligases

Small-molecule inhibitors that target the catalytic activity of HECT E3 ligases or disrupt their interactions with substrates are being developed as potential cancer therapeutics. For example, inhibitors targeting the interaction between NEDD4-1 and PTEN are being investigated as a strategy to restore PTEN levels and inhibit the PI3K [45,208,209]. Similar approaches can be used to target TRIP12, which is required for efficient proteasomal degradation of tumor suppressor SCF-(FBW7) ligase. However, one major challenge in designing small-molecule inhibitors against the HECT family of E3 ligases is the specificity issue arising from highly conserved family members. A previous effort to design inhibitors against this family of E3 ligases led to the identification of pan-HECT domain inhibitors with varying degrees of affinity towards most of the HECT-domain family members [210].

## 22. Small-Molecule Inhibitors of RING E3 Ligases

Some of the most prominent small-molecule inhibitors targeting RING E3 ubiquitin ligases are as follows:

*Nutlin-3*: It is the first-generation inhibitor of E3 ubiquitin ligase MDM2 and TP53’s interaction [211]. This small-molecule inhibitor disrupts the interaction between MDM2 and TP53, stabilizing and activating TP53. Although Nutlins have shown good cellular activity, their poor pharmacokinetic properties limit their application for clinical development. A second-generation Nutlin family member, RG7112, retains most of the structural features of Nutlins and has shown improved biochemical activity and selectivity [212]. In addition, several Nutlin derivatives have shown efficacy in preclinical models of MDM2-overexpressing cancers, and clinical trials are ongoing to evaluate their potential in patients [213].

*XIAP inhibitors*: X-linked inhibitor of apoptosis E3 ubiquitin ligase (XIAP) is a multifunctional protein with several domains and an E3 ubiquitin ligase activity. It is a major inhibitor of the apoptosis pathway, and it directly inhibits several caspases and blocks cell death [214]. The activity of this E3 ligase is upregulated in many cancers. At least two different inhibitors have been developed against XIAP, which have shown promise in preclinical settings. Genetic and pharmacological inhibition of XIAP sensitizes cancer cells to various chemotherapeutics [215]. AEG3516 (GEM640) is a second-generation antisense oligonucleotide specifically targeting XIAP, which has shown promise in clinical trials [216]. More recently, LCL161, a generic IAP inhibitor, was developed to target tyrosine kinase inhibitor-resistant leukemia cells [217]. LCL161 is a second mitochondrial-derived activator of caspases (SMAC) mimetic, which prevents the binding of SMAC with the IAPs, including the XIAP, and enhances cellular apoptosis [217]. AEG3516 and LCL161 are being tested in clinical trials for some form of leukemia, but their efficacy in solid cancers needs to be tested.

*BI8622 and BI8626*: These compounds were identified in a chemical biology screen to inhibit E3 ligase HUWE1’s ubiquitination activity [218]. They selectively block MYC-mediated transcriptional activation by stabilizing the global repressor of MYC proteins, MIZ, in a tumor cell-specific manner [218]. These compounds have also shown promise in preclinical MM models by reducing the growth of MM cell lines and patient-derived cell lines [219].

*MLN4924*: The cullin–RING ligase complex assembly requires the neddylation of its CUL partner by the NEDD8-activating enzyme (NAE). MLN4924 interferes with NAE activity and blocks the transfer of NEDD8 on the cullin ligases, leading to inhibition of this complex and stabilization of numerous CRL substrates, including c-MYC, c-JUN, CyclinE, MCL1, and NOTCH proteins. This accumulation of CRL substrates induces catastrophic death and apoptosis of cancer cells. It is currently being evaluated in clinical trials for various cancers, including leukemia, lymphoma, and solid tumors [220].

*SPOP Inhibitors*: SPOP regulates ubiquitination-mediated proteasomal degradation of PD-L1. This brings SPOP into the limelight as a target to synergize cancer immune therapy. Palbociclib, a CDK4/6 inhibitor, enhances SPOP degradation and improves anti-tumor immunity in in vivo models. Palbociclib is being tested in multiple clinical trials for solid tumors [221,222]. It will be interesting to see if palbociclib treatment in combination with immune checkpoint inhibitors will result in lasting cancer immunity in some of those patients. Another SPOP inhibitor has shown promise in pre-clinical renal cell carcinoma models [223]. However, the utility of this inhibitor is yet to be tested in other cancers.

*Other inhibitors*: Competitive inhibitors of ligase substrate interaction targeting other cullin–RING ligase complexes, including β-TRCP [224], FBW7 [225], and SKP2 [226], have been developed. These small molecules have shown anti-cancer activities in vitro cellular models, but as with SPOP inhibitors, further research is needed to establish their anti-cancer activity in in vivo models.

## 23. Proteolysis-Targeting Chimeras (PROTACs)

PROTACs are an innovative class of small molecules that facilitate targeted protein degradation. Unlike traditional inhibitors that merely obstruct the activity of a protein, PROTACs utilize the cell’s UPS to remove the target protein [227,228]. A PROTAC is a dual-role molecule that binds to both an E3 ligase and a target protein, bringing them close together and prompting ubiquitination and degradation of the target protein [227,228]. This method enables the specific degradation of oncogenic proteins that are otherwise difficult to target with small molecules. This property of PROTACs can be specifically exploited in situations where traditional inhibitors have failed due to resistance or in cases where the function of a protein needs to be completely abrogated.

The last decade has witnessed a boom in the PROTAC field, and several PROTAC molecules have been developed. Many of those PROTACs have shown promise in multiple cancer models. Some of the most prominent ones are as follows:

*ARV-110*: This PROTAC is specifically designed to degrade the androgen receptor (AR), which is crucial in driving prostate cancer [229]. In cases of advanced prostate cancer, patients develop resistance to standard androgen deprivation therapies (ADTs) due to *AR* mutations, leading to the continued growth of cancer cells. Notably, ARV-110 can effectively degrade both wild-type and mutant forms of AR, thereby inhibiting tumor growth even in cases of resistance. This compound is presently undergoing clinical trials for patients with metastatic castration-resistant prostate cancer (mCRPC) who have become resistant to other therapies [230]. Encouragingly, early trial results have demonstrated significant reductions in AR protein levels and anti-tumor activity in patients [230].

*ARV-471*: This PROTAC targets the ER for degradation, which is crucial for the growth of ER+ breast cancers [231]. Unlike selective estrogen receptor degraders (SERDs), which only inhibit ER activity, ARV-471 leads to the complete degradation of the estrogen receptor, preventing estrogen-dependent signaling and tumor growth. ARV-471 is in clinical trials for patients with ER+ breast cancer, particularly those who have developed resistance to endocrine therapies such as tamoxifen and aromatase inhibitors. Early results show that ARV-471 has potent anti-tumor effects and is well tolerated [232,233,234].

*DT2216*: BCL-XL is an anti-apoptotic protein that is overexpressed in many cancers, including blood cancers like leukemia and lymphoma. DT2216 is a BCL-XL-targeting PROTAC that induces BCL-XL degradation, promoting apoptosis in cancer cells [235]. DT2216 selectively degrades BCL-XL in cancer cells while reducing the risk of toxicity due to BCL-XL targeting in normal cells [235]. DT2216 is in preclinical development but has shown potent anti-tumor activity in models of leukemia, lymphoma, and certain solid tumors [235,236,237]. This PROTAC is especially promising for overcoming resistance to chemotherapy and other anti-cancer therapies that rely on the induction of apoptosis [236].

*BET PROTACs*: BET proteins, particularly BRD4, are epigenetic regulators involved in transcription and cell cycle control. BET proteins are essential for the expression of oncogenes such as MYC, and their inhibition has shown efficacy in several cancers [124,125]. However, BET inhibitors have limitations, including toxicity and resistance development [238]. BET-targeting PROTACs, such as ARV-825, induce the degradation of BET proteins, suppressing oncogene expression and cancer cell death [239]. These are being explored in preclinical models for cancers like AML, MM, and certain solid tumors [238]. These PROTACs have demonstrated potent anti-cancer activity and the ability to overcome resistance to BET inhibitors [238].

PROTACs specifically targeting BRD4 have also been developed (dBET1 and dBET57). BRD4 is crucial for the expression of genes that promote cell growth and survival, making it an attractive target for cancer therapy. dBET1 induces the degradation of BRD4, suppressing MYC expression and apoptosis in cancer cells [240]. While still in preclinical stages, dBET1 has shown promising results in animal models of AML, suggesting its potential as a therapeutic agent for hematologic cancers [240]. Similarly, dBET57 has also shown anti-proliferative effects in neuroblastoma [241], and it might be useful in other solid cancers.

## 24. Insights from Early Clinical Trials of Small-Molecule Inhibitors

Despite the plethora of studies demonstrating the utility of targeting UPS members in pre-clinical studies against several cancers, very few small-molecule inhibitors have made it to clinical trials. So far, only three to four small molecules have been reported to be tested in clinical trials [213,216,242,243,244]. Among those are derivatives of TP53 degradation inhibitor Nutlin-3, RING ligase inhibitor MLN4924, and XIAP inhibitors AEG3516 and LCL161, which are tested in clinical trials against certain forms of leukemia with a modest effect on overall patients’ prognosis [213,216,242,243,244]. The FDA-approved cullin–RING ligase inhibitor MLN4924 (pevonedistat), which showed a modest effect in phase I trials of AML patients [244], has shown promising results in multiple phase I trials and is being tested as a combination therapy [245,246,247]. The UBA1 inhibitor TAK-243, which has shown promise in translational studies, was being tested in a phase I clinical trial that is now terminated without any explanation.

Bortezomib (BTZ), the first FDA-approved, broadly acting proteasome inhibitor, treats relapsed refractory MM and mantle cell lymphoma. Pre-clinical studies demonstrated BTZ’s efficacy against various human cancers, including breast, lung, and prostate cancers, both as a single agent and combined with other chemotherapeutic agents in clinical trials [248,249,250,251,252,253]. However, BTZ has dose-limiting toxicity and has a range of side effects, including peripheral neuropathy, fatigue, gastrointestinal issues, myelosuppression, and cardiotoxicity. Thus, caution is needed for the long-term prescription of BTZ.

Many companies, including Arvinas, C4 Therapeutics, Kymera Therapeutics, and Captor Therapeutics, are actively advancing their PROTAC technology. Arvinas is leading the way by initiating phase I trials for their oral PROTAC ARV-110, which targets androgen receptors in mCRPC. Other PROTACs currently in phase I or II clinical trials showed great promise for clinical usage. However, the binding interactions between PROTACs, target proteins, and E3 ligases are based primarily on empirical evidence and lack a robust theoretical foundation. To improve the clinical efficacy of PROTACs, future research should focus on enhanced design techniques using computational approaches, such as structure-based design and artificial intelligence, to optimize ligand–target interactions. Developing novel PROTAC variants with more selective ligands and E3 ligases can aid in reducing off-target effects.

## 25. Challenges in Pharmacological Targeting of UPS Family Members

Several small-molecule inhibitors have been reported and tested in preclinical models. However, the development of these small molecules poses certain challenges, making the transition of these inhibitors from bench to bedside relatively slow. Specifically, targeting E3 ubiquitin ligases presents several challenges due to these enzymes’ structural and functional complexities. Unlike traditional enzymes like proteases or kinases, E3 ligases often do not have a well-defined active site to which small molecules can easily bind. Many E3 ligases facilitate interactions between proteins, such as binding to the E2 ubiquitin-conjugating enzyme and the substrate protein. These interactions typically involve large, shallow interfaces that are not easily targeted by small molecules. This makes it difficult to design inhibitors that can precisely block these protein–protein interactions without affecting other processes within the cell. Moreover, the distinct families of E3 ligases mean they are a highly conserved group of enzymes, and targeting a single enzyme without affecting other family members is nearly unrealistic. Thus, there is a significant risk of unwanted off-target effects.

Another significant challenge posed in the pharmacological targeting of UPS members is their tissue-specific function. In hematological malignancies such as MM and lymphoma, the proteasome is essential for cell survival due to the elevated level of protein turnover. Indeed, inhibiting the function of the proteasome to block protein degradation and induce catastrophic cancer cell death has been proposed as a strategy to block cancer growth. However, many proteasome inhibitors, including bortezomib, carfilzomib, and ixazomib, have a narrow therapeutic window, often resulting in adverse side effects ranging from peripheral neuropathy to myelosuppression and cardiotoxicity [11]. This is probably due to the accumulation of misfolded proteins in normal tissues. These off-target effects may cause toxicities and challenges that complicate the therapeutic management of patients. Additionally, there is a relatively high chance of developing resistance to these inhibitors. This often develops due to overexpression of proteasome subunits, mutations in binding sites, and activation of alternative signaling pathways, leading to multidrug resistance in cancer therapy [11]. This suggests that proteasome inhibitors may be less effective in solid tumors due to their reliance on distinct UPS components [254].

Similarly, FBW7 and GSK3α-mediated inhibition of the NF-κB pathway is a pro-survival mechanism in MM. At the same time, loss-of-function *FBW7* mutations are a hallmark of chemotherapy resistance and poor patients’ prognosis in multiple solid tumors [255,256]. The DUB, USP28, has a dual function in cancer biology, and its role as an oncogene or tumor suppressor is debatable [257]. USP9X stabilizes the anti-apoptotic MCL1 protein and promotes myeloma cell survival, and its inhibition is proposed to sensitize cancer cells to chemotherapy [258]. However, USP9X is a tumor suppressor in both the colon and pancreas [54,105], making USP9X inhibition unsuitable. Thus, careful consideration is needed when designing inhibitors against some of the UPS members, as they well might have tissue-specific opposing functions.

The emergence of PROTACs has revolutionized the approach to targeting “undruggable” proteins that traditional small molecules cannot effectively inhibit. Unlike conventional drugs, which rely on binding to active pockets, PROTACs employ a novel approach by binding to an accessible region or crevice on target proteins, thereby achieving therapeutic effects by degradation of entire proteins rather than just inhibiting their function. However, despite extensive optimization efforts, peptide-based PROTACs faced several inherent limitations, such as high molecular weight, limited efficacy, poor cell permeability, and peptide bond lability, which restricted their clinical applicability. Furthermore, the lack of specificity resulted in PROTAC targeting raised concern about potential off-target effects, resulting in unintended protein degradation in healthy tissues.

In recent years, significant efforts have been made to improve the targeting specificity of PROTACs, such as antibody-based targeting chimeras (AbTACs), optically controlled PROTACs, folate-caged PROTACs, aptamer-PROTAC conjugates (APCs), and others [259]. These advancements improve targeting precision, stability, and on–off capabilities, allowing for spatial and temporal control over protein degradation, recruiting new E3 ligases, and achieving their desired effects with reduced toxicity to normal cells [259].

Finally, in-depth mechanistic studies are required to understand how target protein degradation affects cellular responses and immune interactions. Using bioluminescence resonance energy transfer (BRET) technology to monitor interactions and degradation pathways in live cells holds significant promise for future PROTAC research [227]. Lastly, identifying biomarkers that predict patient responses to PROTACs would enable personalized therapy.

## 26. Conclusions

With approximately 1000 components, the UPS is a crucial protein group that regulates protein homeostasis in physiology and diseases. Several members of the UPS are found to be dysregulated in various human cancers, making them interesting targets for therapeutic intervention. Despite significant progress in understanding the role of E2/E3 ubiquitin enzymes, only a handful have been fully characterized. Given the broader role of E3 ubiquitin ligases in physiology, understanding the functional biology of this class of proteins will pave the way for innovative approaches to treating cancer and other diseases. However, a concerted effort is needed to overcome the challenges in targeted therapies, specifically overcoming the specificity and resistance issues and expanding the range of targetable ligases.

Moreover, protein ubiquitination is a fundamental regulatory mechanism cancer cells exploit to evade the immune system. Tumors can hijack cellular ubiquitination machinery to downregulate antigen presentation, inhibit immune cell activation, induce immune cell apoptosis, and create an immunosuppressive microenvironment. Understanding these mechanisms will also open new opportunities for therapeutic intervention, particularly in the context of immunotherapy. Targeting the ubiquitin–proteasome system could enhance the anti-tumor immune response and improve patient outcomes.

## Figures and Tables

**Table 1:** Summary of prominent HECT E3 ligases and their biology in cancer pathogenesis.

HECT E3 Ligase	Cancer-Related Protein Targets	Related Biological Pathway	Alterations in Cancer	Proposed Action
E6-AP	Tumor suppressor p53 (Tp53)	Apoptosis	Overexpression	Oncogene
HUWE1 (ARF-BP1)	Tp53, c-MYC, and MCL-1	DNA damage, cell fate, and apoptosis	Loss-of-function mutations and homozygous deletion	Tumor suppressor
NEDD4-1	PTEN, MDM2, and Notch	Cell viability and protein localization	Overexpression	Oncogene
ITCH	c-Jun, JunB, and Notch	Immune response	Overexpression	Oncogene
SMURF1/2	TGF-β pathway proteins	Cell polarity and migration	Amplification and overexpression	Oncogene
WWP1	p63, p73, and TGF- βRI	Cell growth, autophagy, and protein localization	Amplification, overexpression and hyperactivating mutations	Oncogene
WWP2	PTEN, p73, Smad2, Smad3, PITCH1	DNA damage, cell fate, and apoptosis	Overexpression and hyperactivating mutations	Oncogene
TRIP12	RNF168, FBW7, and USP7	Cell cycle, DNA damage, and Chemotherapy resistance	Amplification, deletion, and mutations of unknown significance	Oncogene?
UBR5	Groucho	Protein quality control, metastasis, and WNT signaling	Amplification	Oncogene

**Table 2:** Summary of some of the prominent RING E3 ligases and their biology in cancer pathogenesis.

RING E3 Ligase	Cancer-Related Protein Targets	Related Biological Pathway	Alterations in Cancer	Proposed Action
CUL1^FBW7^	Cyclin E, c- Myc, c-Jun	Cell cycle regulation, DNA double-strand break (DSB) repair, DNA replication, apoptosis	Overexpression	Oncogene
CUL2^VHL^	HIF-1α, EGFR	Hypoxia response, angiogenesis, cell signaling pathways.	Downregulated or reduced expression	Tumor suppressor
CUL3_KEAP1_	KEAP1, NRF2	Oxidative stress response, cellular defense mechanism	Overexpression/loss-of-function mutations	Oncogene/tumor suppressor
CUL3_SPOP_	BET	Transcription regulation	Loss-of-function mutations	Tumor suppressor/oncogene
CUL4A^SKP2^	p21, p27	Cell cycle regulation	Overexpression	Oncogene
BRCA1/BARD1	RPB1, CtIP, Cyclin B1, Histone H2A	DNA repair, genome stability	Germline mutations, loss of function	Tumor suppressor
Mdm2	P53	Cell cycle regulation, DDR, and apoptosis	Overexpression or amplification	Oncogene
RNF168	Histones (H2A, H2AX)	DNA damage response, genome stability	Loss-of-function or dysfunction in RNF168	Tumor suppressor
PARKIN	FBP1, β-catenin, Hsp70, p38	Mitophagy dysfunction, stress response.	Mutations, loss of function	Tumor suppressor

## Data Availability

Not applicable.
